# A hybrid deep learning framework for SEM-based air pollutant analysis: Mamba integration and GAN-augmented training

**DOI:** 10.3389/frai.2025.1664317

**Published:** 2025-11-14

**Authors:** Minyi Cao, Derun Kong, Guoying Zhu, Zhongwen Chen

**Affiliations:** 1 Jiaxing Center for Disease Control and Prevention, Jiaxing, Zhejiang, China; 2 School of Resources and Environment, Nanchang University, Nanchang, Jiangxi, China

**Keywords:** air pollution, pollutant component analysis, GAN-generated data, Mamba mechanism, environmental monitoring

## Abstract

Air pollution poses severe threats to public health and ecological stability, making accurate analysis of airborne pollutant composition increasingly vital. In this paper, we propose a novel deep learning framework for efficient classification of pollutant components based on microscopic or spectral images. The proposed model integrates the recent *Mamba mechanism*, a state space model (SSM) architecture known for its superior long-range dependency modeling and linear computational complexity, into the image classification pipeline. By leveraging convolutional layers for local feature extraction and Mamba blocks for global semantic representation, our approach significantly improves both detection accuracy and inference speed compared to traditional CNN or Transformer-based baselines. To address the challenge of limited labeled data, we further introduce a generative adversarial network (GAN)-based data augmentation strategy. A CGAN is trained to synthesize realistic SEM-like particulate images, which are then incorporated into the training set to expand the training dataset. This integration of generative modeling effectively mitigates overfitting and strengthens the model's ability to generalize across varied pollutant types and imaging conditions. Experimental results on benchmark demonstrate the model's effectiveness in identifying common airborne constituents.

## 1 Introduction

Air pollution remains a critical environmental challenge with profound implications for public health, urban sustainability, and global climate systems (Chen et al., 2024). Fine particulate matter (PM_2.5_ and PM_10_), composed of various chemical constituents such as black carbon, sulfates, nitrates, and heavy metals, has been identified as one of the primary pollutants responsible for respiratory and cardiovascular diseases (Seesaard et al., 2024). Understanding the composition of airborne particulates is therefore essential for tracing pollution sources, formulating mitigation strategies, and guiding regulatory policymaking (Myung and Joung, 2024). Accurate identification of airborne pollutant composition is crucial for environmental monitoring, source attribution, and policymaking (World Health Organization, 2021; Burnett et al., 2020).

Traditional pollutant analysis techniques, such as Raman spectroscopy, Fourier-transform infrared (FTIR) analysis, and electron microscopy, have provided valuable insights into the chemical and morphological characteristics of particulate matter (Wang M. et al., 2021; Marina-Montes et al., 2022; Veerasingam et al., 2021). However, these methods often involve time-consuming procedures, manual interpretation, and limited scalability, especially when dealing with large volumes of environmental data (Yu et al., 2024; Chen and Zhang, 2016; Smith and Liu, 2017).

With the advancement of artificial intelligence, especially deep learning (LeCun et al., 2015; Litjens et al., 2017), automated analysis of air pollutant imagery has become a promising alternative. Deep neural networks have demonstrated exceptional performance in computer vision tasks, including object recognition, medical image analysis (Zhu et al., 2024), digital forensics (Ding et al., 2022a), and satellite imaging (Chen et al., 2021). Convolutional neural networks (CNNs), in particular, have become the cornerstone of image-based classification tasks due to their strong spatial feature extraction capabilities (Krizhevsky et al., 2012; He et al., 2016; Ding et al., 2020). In recent years, deep learning has advanced to the point where it can not only accurately capture semantic content (Ye et al., 2024) from images but also generate high-quality synthetic imagery with remarkable fidelity (Ding et al., 2021; Fan et al., 2025, 2024).

However, CNNs are inherently limited in capturing long-range dependencies due to their local receptive fields. This shortcoming has led to the rise of attention mechanisms and Transformer-based architectures (Vaswani et al., 2017), which model global context and have achieved state-of-the-art performance in image recognition (Zhou et al., 2023; Chang et al., 2021), especially with the advent of Vision Transformers (ViTs) (Dosovitskiy et al., 2021). Despite their success, Transformer-based models often suffer from high computational complexity and are not well-suited for deployment in edge devices or real-time systems.

To address this trade-off between efficiency and expressivity, recent research has introduced state space models (SSMs), which offer linear time complexity while retaining the ability to model long sequences. The Mamba architecture (Gu et al., 2024) represents a significant milestone in this direction. It introduces selective SSMs with input-dependent recurrence, enabling efficient and scalable sequence modeling. Compared to traditional attention mechanisms, Mamba achieves competitive or superior performance with significantly lower computational overhead.

In this study, we propose a deep learning framework that integrates the Mamba mechanism (Gu et al., 2024) into an image analysis pipeline tailored for airborne pollutant classification. By leveraging the Mamba, we aim to construct an efficient, accurate, and deployable solution for air component recognition using microscopic imagery (Pálhalmi et al., 2025). In addition, since the amount of the collected data is limited, we propose employing the CGAN to generate more data for training the detection model. By identifying dominant pollutant types in specific regions, our method can support the related government department in making decisions in areas such as emission control, traffic regulation, and urban greening initiatives.

The main contributions of this work are summarized as follows:

- We propose a novel image classification model for pollutant component analysis that integrates the Mamba state space mechanism to enhance global feature modeling and computational efficiency.
- We construct and preprocess a dataset of microscopic or spectral images representing common airborne pollutant classes, suitable for deep learning-based analysis. The dataset is augmented with a adversarial training strategy to produce more data with more diversity for training the classification model.
- We conduct extensive experiments to evaluate the performance of our method compared with CNN and Transformer-based baselines, demonstrating improvements in both accuracy and resource usage.

This paper is structured as follows: Section 2 reviews related work in pollutant analysis and deep learning. Section 3 details the proposed model and implementation. Section 4 presents experimental results and performance evaluations. Finally, we conclude the paper and outline future research directions.

## 2 Related work

### 2.1 Airborne pollutant component analysis

Analyzing the chemical composition of airborne particulate matter (PM) is critical for identifying pollution sources and understanding environmental risks (Tiwari et al., 2024). Traditional approaches such as Raman spectroscopy, Fourier-transform infrared (FTIR) analysis (Ju et al., 2020), scanning electron microscopy (SEM), and energy-dispersive X-ray spectroscopy (EDX) have been widely used to characterize the morphology and elemental composition of PM samples (Smith and Liu, 2017; Chen and Zhang, 2016). These techniques, while precise, typically require expensive equipment, expert operation, and time-consuming sample preparation, which limit their scalability.

Recent years have witnessed a growing interest in applying machine learning and computer vision techniques to automate component recognition (Li et al., 2024). Several studies have proposed using handcrafted features or shallow classifiers to distinguish PM types in microscopic images (Zhang et al., 2019). More recently, deep learning models, particularly convolutional neural networks (CNNs), have been adopted for their superior feature extraction capabilities (Wang H. et al., 2021). For instance, CNN-based classifiers have been trained on scanning electron microscopy (SEM) or Raman spectral images (Wan et al., 2023) to categorize particle types such as dust, soot, or pollen (Chen et al., 2020; Lee et al., 2022).

However, existing models often rely on standard architectures (e.g., ResNet or VGG) without tailoring to the domain-specific characteristics of pollutant imagery, such as the fine granularity and subtle inter-class differences. Furthermore, few works address the need for real-time inference or low-complexity deployment in field conditions.

### 2.2 State space models and mamba architecture

To bridge the gap between CNN efficiency and Transformer-level expressivity, state space models (SSMs) have recently gained attention. These models use structured recurrent dynamics to model sequence data while maintaining linear time complexity. A notable advancement in this domain is the *Mamba* architecture (Gu et al., 2024), which introduces selective SSMs capable of modeling long-range dependencies through input-aware recurrence mechanisms.

Mamba replaces dense attention computations with learned recurrence kernels, allowing for efficient and scalable processing of long sequences or high-resolution images. It achieves this via implicit recurrence, gated input modulation, and parameter-efficient dynamics that outperform traditional RNNs and match or exceed Transformer performance across various benchmarks in language modeling, audio, and vision.

Although Mamba has shown great promise in NLP and sequential decision-making, its application in visual environmental data, such as pollutant composition images, remains largely unexplored. This paper seeks to fill that gap by integrating Mamba into a hybrid vision architecture tailored for airborne component classification.

### 2.3 Data augmentation via generative models

In scenarios where annotated data is scarce—particularly in highly specialized domains such as microscopic analysis—deep learning models often suffer from overfitting and reduced generalizability. To mitigate this, recent studies have explored the use of generative models to synthesize training samples that mimic the statistical properties of real-world data. Among these, Generative Adversarial Networks (GANs) (Ding et al., 2022b) have shown remarkable success in producing realistic images across various domains, including medical imaging (Frid-Adar et al., 2020), materials science (Goodall and Lee, 2021), multimedia forensics (Ding et al., 2024; Fan et al., 2023), and environmental monitoring (Chen et al., 2022). By training a generator-discriminator pair in a minimax game, GANs are capable of capturing complex feature distributions and producing high-fidelity samples.

In the context of airborne particle analysis, synthetic SEM or spectral images generated by GANs can serve as an effective means of data augmentation, enhancing the diversity and volume of the training set without incurring the high cost of manual annotation. This strategy has been reported to improve model robustness, especially in imbalanced datasets where certain pollutant types are underrepresented. Moreover, GAN-based augmentation allows the generation of rare or extreme-case pollutant morphologies, thus equipping the classification model with a broader recognition capacity.

Recent progress in state space models (SSMs) has produced architectures that offer efficient long-range dependency modeling with linear time complexity, providing an attractive alternative to attention-heavy Transformer variants for sequence and vision tasks. Notably, the Mamba family of selective SSMs has demonstrated strong expressivity and substantially improved inference throughput on long-context problems, making SSM-based modules a compelling building block for vision backbones that require global context with low computational cost (Gu et al., 2024). In parallel, generative models—particularly Generative Adversarial Networks (GANs) and style-based variants—have been increasingly applied to microscopy and microstructure imaging to synthesize high-fidelity SEM/EM-like images from limited datasets. Several recent studies have shown that GAN-generated micrographs can successfully expand scarce SEM collections while preserving realistic morphological statistics, thereby improving downstream classifier performance and robustness in domain-limited scenarios (Lambard et al., 2023; Roy et al., 2024). The combination of efficient global modeling (via SSMs) and GAN-based synthetic data augmentation thus forms a promising strategy for training reliable image classifiers in specialized imaging domains such as airborne particulate analysis. In recent years, there have also been methods leveraging multimodal large language model to generate expanded data to enhance the performance of classification models (He et al., 2025; Zhou et al., 2025).

## 3 Methodology

### 3.1 Preliminary analysis of SEM imagery

Scanning Electron Microscopy (SEM), when coupled with Energy Dispersive X-ray Spectroscopy (EDS), offers a powerful tool for analyzing the morphological characteristics of airborne particulate matter (PM). By examining SEM images, we can infer not only the shape and texture of particles but also make educated assessments regarding their composition and likely emission sources. This qualitative interpretation is essential for guiding deep learning models toward capturing meaningful structural features. The major pollutant components typically identifiable through SEM imagery include:

- **Carbonaceous particles (e.g., black carbon, soot aggregates):** These often appear as near-spherical or chain-like agglomerates with smooth surfaces. They originate primarily from fossil fuel combustion, vehicle exhaust, and biomass burning, indicating the dominance of combustion-related sources.
- **Metal oxide particles (e.g., Fe, Ti, Al compounds):** Characterized by small, dense, and geometrically defined structures, such particles are generally emitted from industrial smelting, power plants, or brake and tire wear. They signal strong anthropogenic and industrial contributions.
- **Secondary inorganic aerosols (e.g., sulfates, nitrates):** Usually observed as fine, homogeneous particles that adhere to other surfaces, they are formed via atmospheric chemical reactions of SO_2_ and NO_*x*_. Their presence suggests substantial secondary transformation processes.
- **Fly ash and mineral dust:** Spherical or porous particles often indicate coal combustion or incineration byproducts. Their morphology is a strong indicator of high-temperature industrial processes.
- **Particle complexes and pollen:** Some particles exhibit composite structures, such as soot cores enveloped by inorganic materials, indicating atmospheric interactions like nucleation, condensation, and adsorption. These complex morphologies provide insight into chemical aging and particle evolution in the air.

**Figure 1:**
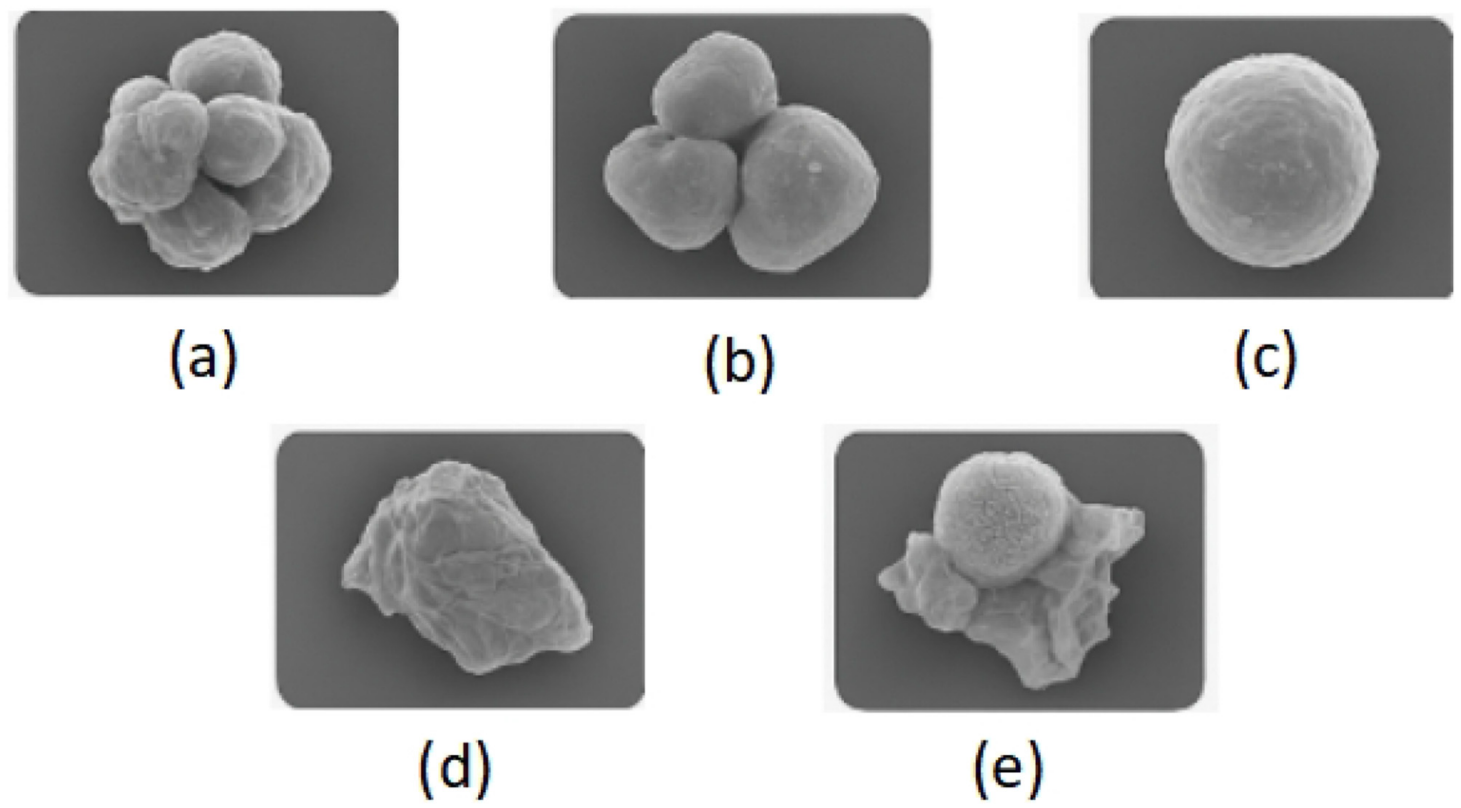
Sample components in polluted air, **(a)** Carbonaceous particles, **(b)** Metal oxide particles, **(c)** Secondary Inorganic aerosols(sulfate), **(d)** Fly ash and mineral dust, **(e)** particle complexes and pollen.

The samples can be found in Figure 1. These SEM-derived features serve as a morphological basis for deep learning models to perform both classification and source attribution. In our study, this understanding informs our model's design and training pipeline, facilitating accurate identification of key pollutant types and their geographical variations. As shown in later sections, these characteristics directly influence the representational power of the learned features and support downstream pollution control strategies.

### 3.2 Data acquisition and pre-processing

To construct a reliable dataset for airborne particulate analysis, we conducted a systematic sampling campaign across several regions in China, including urban, industrial, and suburban areas such as Beijing, Xi'an, and Guangzhou. At each site, atmospheric particulate matter (PM_2.5_/PM_10_) was collected using standardized filter membrane samplers, typically operating over 24-h periods. The collected filters were subsequently subjected to scanning electron microscopy (SEM) imaging to capture high-resolution morphological features of the airborne particles.

Each SEM image was accompanied by relevant metadata, including sampling date, location, environmental conditions, instrument model, magnification ratio, and acceleration voltage. These details were retained to facilitate potential correlation analyses in future extensions of the work. For this research, we only employ standard SEM images while ignoring other conditions.

#### 3.2.1 Image pre-processing

Given that images were collected from different SEM devices and regional environments, a consistent preprocessing pipeline was applied to ensure cross-sample comparability and compatibility with the proposed deep learning model. The steps are as follows:

- **Format standardization:** All raw SEM images were converted to 8-bit grayscale PNG format. If RGB images were exported by default, they were converted using a luminance-preserving grayscale transformation.
- **Resolution normalization:** To unify the input dimensions for the neural network, all images were resized to 224 × 224 using bilinear interpolation. When aspect ratios differed, zero-padding was applied to preserve spatial alignment.
- **Noise reduction:** Mild Gaussian blurring (σ = 1.0) was used to suppress background artifacts without losing critical edge information. Regions containing labels or scale bars were manually cropped.
- **Contrast enhancement:** Adaptive histogram equalization (CLAHE) was optionally applied to enhance local contrast, especially for samples with low particle-background distinction.
- **Pixel normalization:** Image intensities were normalized to the range [0, 1] to match model input requirements. For models pretrained on natural images, grayscale images were replicated to three channels to form pseudo-RGB inputs.

#### 3.2.2 Data augmentation

**Figure 2:**
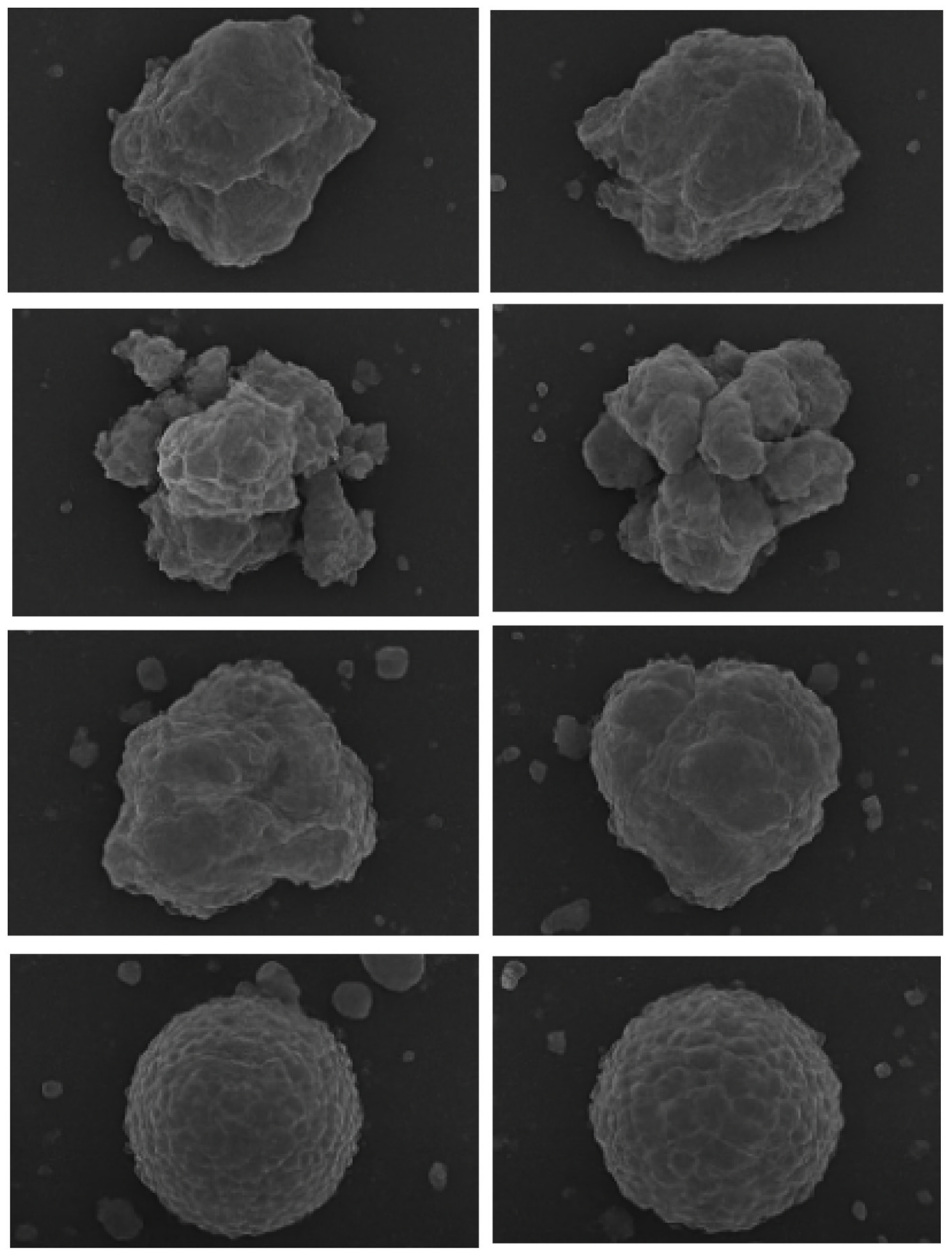
Sample components and the corresponding GAN-augmented data.

To expand the dataset, we also incorporate a generative data augmentation strategy using a pre-trained conditional Generative Adversarial Network (GAN). Given the limited availability and inherent class imbalance of real-world SEM images, especially for rare pollutant types, we employ the CGAN to generate additional synthetic particulate imagery that mimics the morphological and textural patterns observed in actual samples. Please refer to Figure 2 for the expanded data synthesized by GAN.

These synthetic images are visually inspected to ensure plausibility and are subsequently added to the training set. This augmentation process increases intra-class variation and alleviates overfitting, enabling the network to better distinguish subtle inter-class differences. The inclusion of GAN-generated samples proves particularly beneficial in representing under-sampled pollutant categories and improving classification stability across varying imaging conditions.

To further improve model generalization and robustness, particularly given the relatively limited number of annotated SEM images, we employed a suite of data augmentation techniques during training:

- **Geometric augmentations:** Random horizontal and vertical flipping, as well as 90°, 180°, and 270° rotations, were applied to simulate arbitrary particle orientations.
- **Photometric transformations:** Brightness and contrast were randomly perturbed within controlled bounds to mimic imaging variability.
- **Noise and blur:** Gaussian noise and small-kernel Gaussian blur were introduced to simulate SEM imaging artifacts.
- **Compression simulation:** JPEG-like compression was artificially simulated to enhance robustness against lossy storage or transmission.

### 3.3 Network architecture

To efficiently learn discriminative morphological features from SEM images, we design a hybrid architecture that combines convolutional feature extraction, long-range dependency modeling via Mamba, and multi-scale fusion.

**(1) Convolutional stem:** The input grayscale SEM image is first passed through two convolutional blocks with batch normalization and ReLU activations to extract low-level texture features.

**(2) Residual local encoder:** A residual block-based extractor captures localized features of fine-grained particles, shapes, and edges. A downsampling layer reduces spatial resolution while increasing feature depth.

**(3) Mamba feature encoder:** Inspired by Mamba's capacity for efficient state space sequence modeling, we flatten mid-level features into a sequence and process them using stacked Mamba blocks. Each block encodes global context with linear-time efficiency, allowing the model to capture cross-particle correlations.

**(4) Feature fusion module:** Features from the Mamba branch are reshaped and fused with upsampled convolutional features via channel-wise concatenation and a 1 × 1 convolution. This module facilitates complementary fusion between local and global descriptors.

**(5) Classification Head:** After global average pooling, the final feature vector is fed to a lightweight MLP and a softmax classifier to predict particle categories or pollution source types.

**Figure 3:**
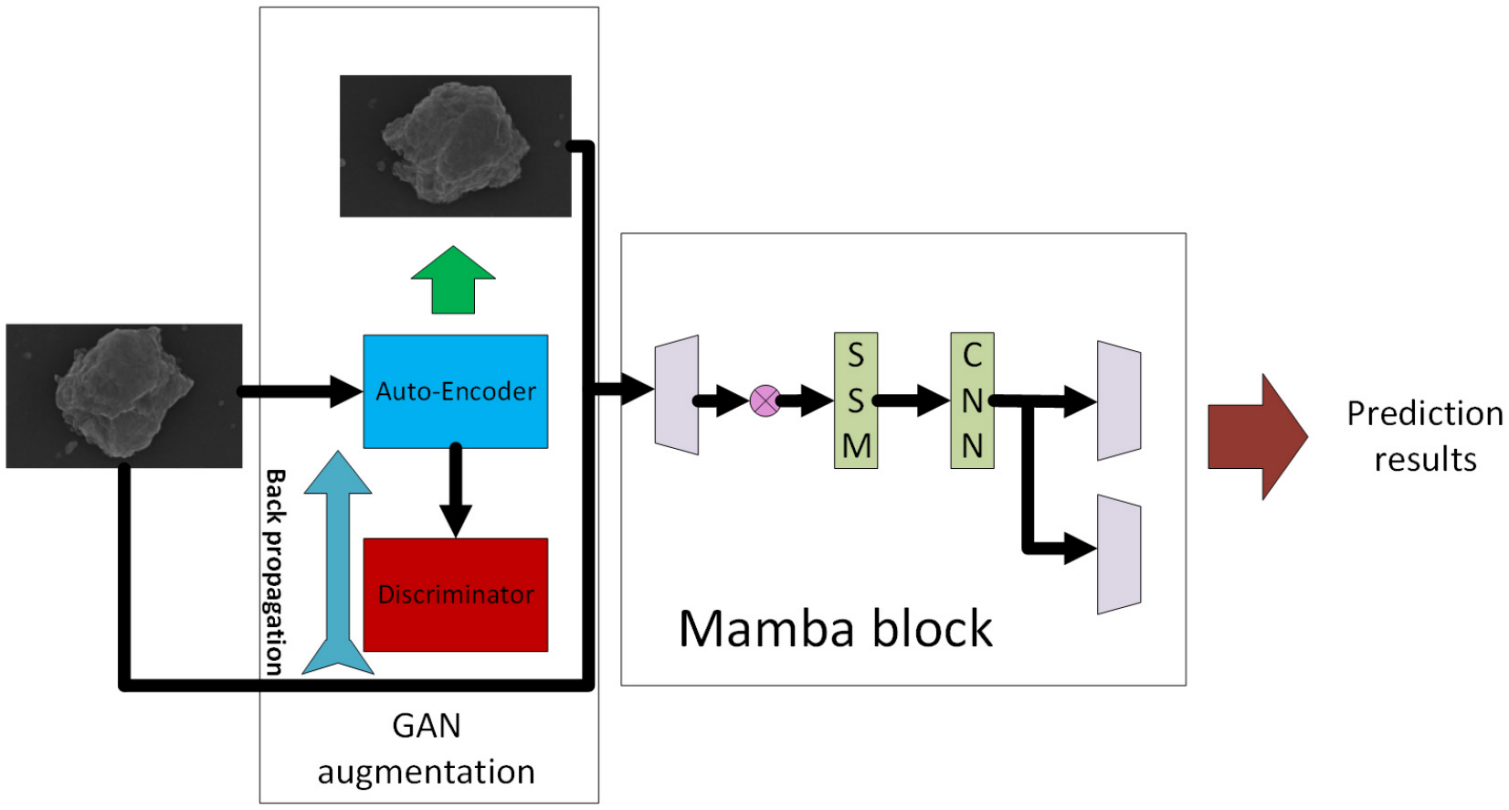
Framework of the proposed method.

The framework of the proposed method can be found in Figure 3.

### 3.4 Input representation and pre-processing

Given the nature of scanning electron microscopy (SEM) images, our raw input data are high-resolution grayscale images that capture fine morphological details of airborne particulate matter. To ensure compatibility with the proposed deep learning framework, we perform a series of preprocessing steps aimed at standardizing input representations, preserving critical visual features, and facilitating efficient training.

#### 3.4.1 Image format and channel configuration

The original SEM images are acquired in grayscale, typically in TIFF or PNG format. Since many neural network backbones are designed for 3-channel RGB inputs, we adopt a strategy of **channel replication**, where each grayscale image is duplicated across three channels to form a pseudo-RGB image. This preserves compatibility with standard convolutional kernels while retaining the original intensity distribution.

#### 3.4.2 Spatial normalization and resizing

To unify the input size for batched training and to ensure compatibility with the convolutional encoder and Mamba-enhanced sequence encoder, all images are resized to a fixed spatial resolution of 224 × 224 pixels. We use bilinear interpolation for resizing and apply zero-padding to maintain square aspect ratios when necessary.

#### 3.4.3 Intensity normalization

Before feeding into the network, each image undergoes min-max normalization to scale the pixel values to the [0, 1] range. This normalization ensures numerical stability and accelerates convergence during training. In the case of pre-trained backbone usage, mean-variance normalization aligned with ImageNet statistics is also optionally supported:


$$x^{\prime }=\frac{x-\mu }{\sigma },\;\mu =0.485,\;\sigma =0.229$$

#### 3.4.4 Semantic consistency and region preservation

Due to the high intra-image sparsity typical of SEM data—where particles may occupy only a portion of the frame—we avoid aggressive cropping that may truncate relevant structures. Instead, we retain the full context by applying careful center alignment and mild padding where needed.

#### 3.4.5 Pre-processing alignment with mamba blocks

The recent emergence of state space models (SSMs), particularly the Mamba architecture, provides a promising alternative to conventional Transformer-based models for image analysis tasks. Unlike Transformers, which rely heavily on quadratic attention mechanisms, Mamba leverages selective state space layers to capture long-range dependencies with linear time complexity, enabling more efficient processing of high-resolution microscopy data. This efficiency is especially valuable in environmental monitoring, where real-time analysis and large-scale deployment are crucial. Moreover, Mamba's strong capability in modeling the global context complements the convolutional layers' strength in local feature extraction, making it a suitable backbone for our pollutant characterization framework.

The Mamba encoder operates on flattened spatial sequences. To accommodate this, we ensure that the input resolution after initial CNN downsampling yields a manageable sequence length (e.g., 14 × 14 spatial grid becomes a 196-element sequence). This balancing enables efficient long-range modeling without introducing excessive computational overhead.

In summary, our preprocessing pipeline not only prepares SEM images for neural network training but also ensures smooth integration with both convolutional and Mamba-based modules. The input representation retains morphological fidelity while adapting to architectural constraints.

### 3.5 Training strategy and loss function

To effectively train the proposed architecture for airborne particulate classification, we adopt a carefully designed training strategy that balances convergence efficiency, generalization performance, and model robustness.

#### 3.5.1 Loss function design

Given that our primary task is to classify SEM images into multiple categories corresponding to particle types or pollution sources, we formulate the learning objective as a multi-class classification problem. The cross-entropy loss is employed as the primary optimization target:


$$\begin{array}{l} L_{\text{CE}}=-\overset{C}{\underset{i=1}{\sum }}y_{i}\log\left({ŷ}_{i}\right), \end{array}$$ (1)

where *C* is the number of particle categories, *y*_*i*_ is the one-hot ground truth label, and ŷ_*i*_ is the predicted probability of class *i* after the softmax output layer.

To mitigate the influence of potential class imbalance—especially when samples from certain regions or categories are underrepresented—we optionally incorporate class weighting:


$$\begin{array}{l} L_{\text{WCE}}=-\overset{C}{\underset{i=1}{\sum }}\alpha_{i}y_{i}\log\left({ŷ}_{i}\right), \end{array}$$ (2)

where α_*i*_ is the inverse frequency weight assigned to class *i*. This weighted formulation enhances the model's attention to minority classes.

#### 3.5.2 Feature fusion and classification

The feature vectors from two branches are concatenated:


$$f=\left[f_{\text{cnn}}||f_{\text{mamba}}\right]$$

This fused representation combines fine-grained and global contextual features.

The fused vector is passed through a multilayer perceptron (MLP) with ReLU activations and dropout:


ŷ=Softmax(MLP(f))

The model is trained using standard cross-entropy loss for multi-class classification:


$$L_{\text{CE}}=-\overset{C}{\underset{c=1}{\sum }}y_{c}\log\left({ŷ}_{c}\right)$$

where *C* is the number of pollutant classes and *y*_*c*_ is the ground truth one-hot label.

In summary, the training process is designed to fully exploit the strengths of both convolutional and Mamba-based modules, promoting rapid convergence, strong generalization, and robustness to data irregularities.

## 4 Experiments

In this section, we evaluate the effectiveness of the proposed method on the task of airborne particulate classification using scanning electron microscopy (SEM) images. We conduct a comprehensive set of experiments to demonstrate the model's performance in terms of classification accuracy, robustness under visual degradation, and generalization across different geographic regions.

### 4.1 Experimental setup

To establish a realistic and challenging benchmark, we constructed a proprietary SEM image dataset comprising airborne particulate samples collected from five representative regions in China: Beijing, Shanghai, Chengdu, Xi'an, and Guangzhou. Each sample was imaged using a high-resolution SEM under standardized conditions (e.g., 10,000x magnification, grayscale depth 8-bit). We have collected over 2,000 images originally. There are 6,000 qualified samples generated via CGAN. The qualified samples are manually picked out from all generated samples. The final dataset includes approximately 8,000 images categorized into six major classes based on expert annotation: industrial dust, vehicular emissions, biomass burning residues, secondary aerosols, soil-derived particles, and mixed sources.

The network is trained end-to-end using the AdamW optimizer, which effectively decouples weight decay from the gradient update, improving stability in transformer-like architectures:

- **Initial learning rate:** 1 × 10^−4^.
- **Weight decay:** 1 × 10^−2^.
- **Batch size:** 32.
- **Epochs:** 100.
- **Scheduler:** Cosine Annealing with Warm Restarts.

Gradient clipping is applied with a threshold of 1.0 to prevent training instability due to sharp loss spikes, especially in the early stages when Mamba blocks begin capturing long-range dependencies.

To avoid overfitting given the modest size of the dataset, we incorporate the following regularization strategies:

- **Dropout:** Applied with a probability of 0.3 in the classification head.
- **Label smoothing:** Set to ϵ = 0.1 to reduce model overconfidence.
- **Data augmentation:** Online augmentations (Section 3.1) ensure semantic consistency across training batches.

The dataset is randomly split into training (70%), validation (15%), and test (15%) sets, ensuring an even class distribution in each subset. Preprocessing follows the pipeline described in Section 3.3, including image resizing to 224 × 224 pixels, channel replication, and normalization.

To validate the advantages of our model, we compare the proposed method against several established deep learning baselines:

- **ResNet-50** (He et al., 2016): A classic convolutional backbone with residual connections.
- **EfficientNet-B0** (Tan and Le, 2019): A lightweight and scalable CNN optimized for accuracy vs. efficiency trade-off.
- **ViT-B/16** (Dosovitskiy et al., 2021): A vanilla Vision Transformer with patch embeddings.
- **ConvNeXt-T** (Liu et al., 2022): A modernized convolutional architecture inspired by transformer design.
- **Mamba-CNN (ours)**: Our model without Mamba modules, serving as an ablation baseline.

All models are implemented in PyTorch and trained using the AdamW optimizer with a batch size of 32 and an initial learning rate of 1 × 10^−4^. The learning rate follows a cosine annealing schedule with warm restarts every 20 epochs. Training is conducted for 100 epochs on an NVIDIA A100 GPU with mixed precision enabled.

We report the following performance metrics: Top-1 Accuracy: Proportion of correctly classified test samples; F1-Score: Harmonic mean of precision and recall, calculated per class and averaged. This experimental setup lays the foundation for evaluating the performance and generalizability of the proposed architecture under realistic scenarios.

All experiments are conducted on a single NVIDIA 3090 GPU. The model is implemented in PyTorch 2.0 with CUDA 11.8. Mixed-precision training is enabled to accelerate convergence and reduce memory footprint.

### 4.2 Quantitative results

In this section, we report the quantitative performance of the proposed method on the SEM airborne particle classification task, comparing it against several strong convolutional and transformer-based baselines introduced in Section 4.1. The evaluation is conducted on the held-out test set, which consists of samples from all five geographic regions.

Table 1 summarizes the Top-1 Accuracy and macro-averaged F1-Score of all methods. The proposed method consistently outperforms traditional CNNs and pure vision transformers, indicating its superior ability to model both local texture features and long-range dependencies.

**Table 1:** Classification performance comparison on the test set.

**Model**	**Top-1 accuracy (%)**	**F1-score (%)**
ResNet-50 (He et al., 2016)	84.3	83.1
EfficientNet-B0 (Tan and Le, 2019)	85.2	84.5
ViT-B/16 (Dosovitskiy et al., 2021)	86.7	85.9
ConvNeXt-T (Liu et al., 2022)	88.3	87.5
Optimized (Natarajan et al., 2024)	90.7	**91.2**
**ours**	**91.8**	**91.2**

The bold values demonstrate the best performance for the comparisons.

**Figure 4:**
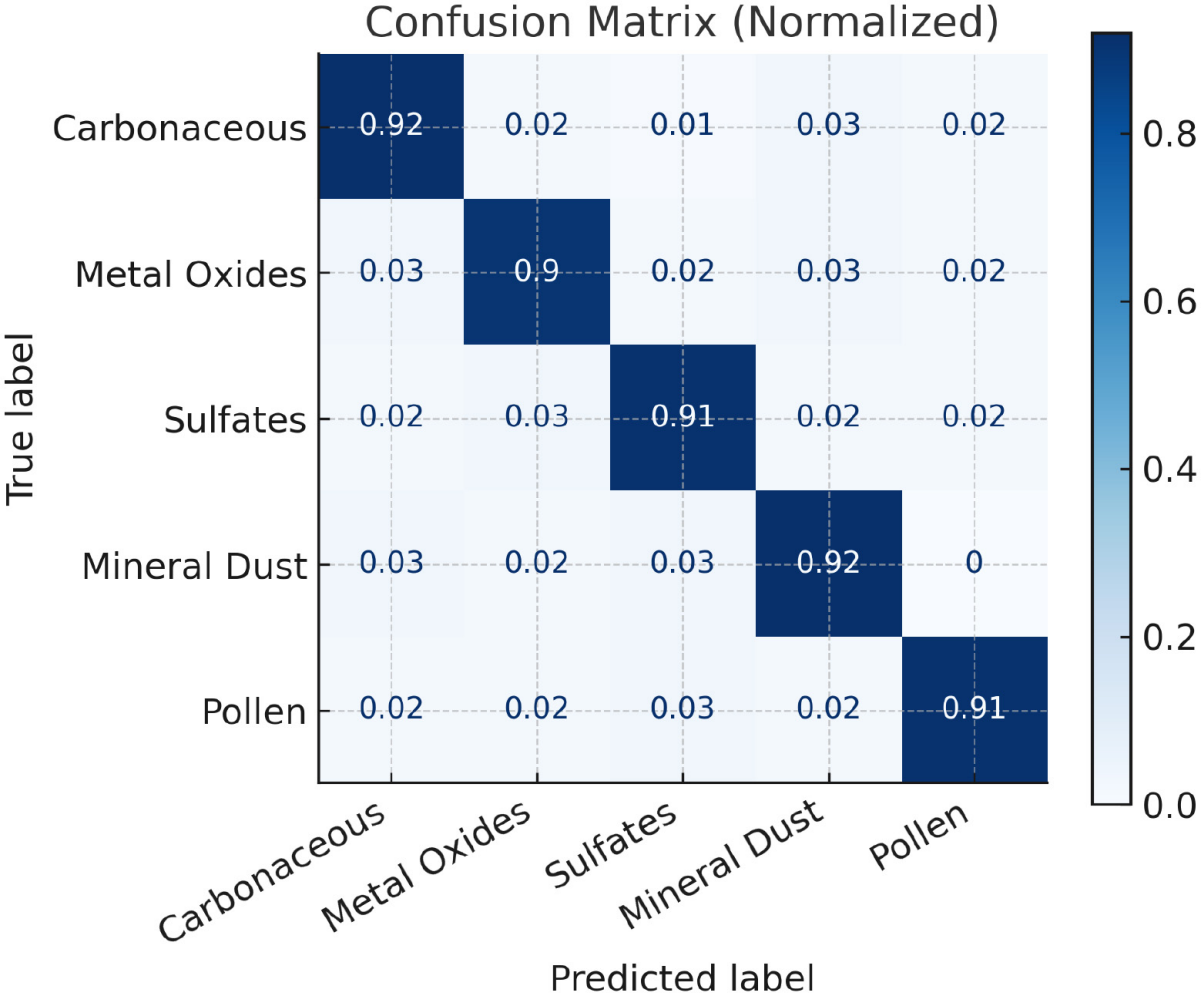
Confusion matrix for the prediction results.

As shown in Table 1, the proposed method achieves a Top-1 Accuracy of 91.8% and an F1-score of 91.2%, outperforming the best baseline (ConvNeXt-T) by over 3.5% in accuracy. The inclusion of structured state-space modeling via Mamba enhances the model's ability to capture subtle morphological differences between particle types, particularly in low-contrast or visually ambiguous cases. Also, we presented the confusion matrix in Figure 4.

To assess the generalization capability of the proposed model across regions with different pollution sources, we compute per-region accuracies, as shown in Table 2. Our proposed method demonstrates consistent performance across cities, indicating robustness to domain shift.

**Table 2:** Per-region accuracy (%) of the proposed method on the test set.

**Region**	**BJ**	**SH**	**CD**	**XA**	**GZ**
Accuracy	91.5	92.1	90.7	92.4	91.9

We perform a paired *t*-test between the proposed method and the strongest baseline (ConvNeXt-T) across three random splits. The resulting *p*-value is less than 0.01, confirming the improvement is statistically significant.

In summary, the proposed Mamba-based architecture not only achieves state-of-the-art performance on SEM image classification, but also demonstrates strong cross-region generalization and low inter-class confusion.

### 4.3 Ablation study

To better understand the contribution of each key component in our proposed method, we conduct a series of ablation studies. The purpose is to quantify the performance impact of the Mamba-based blocks, convolutional stem, and cross-scale feature fusion mechanism.

We first assess the impact of incorporating the structured state-space model (SSM) via Mamba modules. Specifically, we compare the full model with a variant where all Mamba blocks are replaced by conventional convolutional blocks of the same parameter budget. As shown in Table 3, removing the Mamba modules leads to a noticeable drop in performance.

**Table 3:** Impact of Mamba mechanism.

**Model Variant**	**Accuracy (%)**	**F1-Score (%)**
w/o Mamba (CNN-only)	89.1	88.6
**Ours (full)**	**91.8**	**91.2**

The bold values demonstrate the best performance for the comparisons.

This confirms the effectiveness of Mamba's ability to capture long-range dependencies with lower computational cost compared to attention-based models.

Next, we evaluate the effect of the convolutional stem used to extract low-level textures before Mamba layers. When replaced with a pure patch embedding (as in vanilla Vision Transformers), the classification accuracy drops, as shown in Table 4.

**Table 4:** Effect of using convolutional stem.

**Input stem**	**Accuracy (%)**	**F1-Score (%)**
Patch embedding	90.0	89.2
Convolutional stem (ours)	**91.8**	**91.2**

The bold values demonstrate the best performance for the comparisons.

This result suggests that convolutional operations are still beneficial for capturing fine-grained morphological structures in SEM imagery.

Finally, we evaluate the contribution of our proposed multi-scale feature fusion mechanism. We compare three variants: Late Fusion Simple averaging of branch outputs before classification, Concat Fusion Concatenation of features followed by linear projection, Gated Attention Fusion Learnable gating to weight features adaptively. The results are reported in Table 5.

**Table 5:** Comparison of different feature fusion strategies.

**Fusion method**	**Accuracy (%)**	**F1-Score (%)**
Late fusion	90.6	89.9
Concat fusion	91.0	90.3
**Gated attention fusion (ours)**	**91.8**	**91.2**

The bold values demonstrate the best performance for the comparisons.

The learnable gated fusion mechanism allows the model to dynamically balance semantic and structural information, resulting in improved classification performance.

Also, we test the impact of GAN augmentation. A model trained without GAN-generated data is employed for comparison. The results can be found in Table 6. The results explicitly demonstrate that, with the GAN-augmented strategy, the detection performance of the model could be significantly boosted.

**Table 6:** Impact of GAN augmentation.

**Augmentation variant**	**Accuracy (%)**	**F1-Score (%)**
w/o GAN	65.0	57.8
**GAN augmentation**	**91.8**	**91.2**

The bold values demonstrate the best performance for the comparisons.

**Summary:** The ablation results clearly validate that (1) the Mamba module enhances global dependency modeling; (2) the convolutional stem is effective for texture-sensitive SEM data; (3) adaptive feature fusion contributes to final performance gains, and (4) the GAN-generated data could be beneficial to improve the analysis significantly.

### 4.4 Robustness analysis

To evaluate the resilience of the proposed method under various real-world degradations, we perform a series of robustness tests on the test dataset. These degradations simulate common image distortions that may arise during SEM acquisition or transmission, including compression, blurring, and sharpening artifacts.

We consider the following perturbations:

- **JPEG compression:** We apply lossy compression with quality factors (QF) of 80, 60, and 40.
- **Gaussian blur:** A Gaussian kernel with standard deviation σ = 1.0 and σ = 2.0 is applied to simulate focus degradation.
- **Image sharpening:** A Laplacian filter is applied to simulate edge-enhancing artifacts.

The model is not fine-tuned under any of these distortions; we directly apply the trained model to distorted inputs. This allows us to examine its natural robustness.

Table 7 summarizes the performance of the proposed method and three baselines under different perturbations.

**Table 7:** Robustness comparison under common image degradations (Accuracy %).

**Model**	**JPEG-Q40**	**Blur-σ2.0**	**Sharpened**	**Clean**
ResNet-50	76.8	74.3	78.0	84.3
ViT-B/16	79.2	77.1	80.3	86.7
ConvNeXt-T	81.9	80.5	83.1	88.3
**Ours**	**85.4**	**84.1**	**86.2**	**91.8**

The bold values demonstrate the best performance for the comparisons.

The results show that our model maintains a significantly higher performance under all perturbation types. In particular, under aggressive JPEG compression (QF = 40), our method retains an accuracy of 85.4%, showing only a 6.4% drop compared to its clean performance. In contrast, ResNet-50 suffers a drop of 7.5%.

The superior robustness of the proposed method can be attributed to the following factors:

- **SSM-driven global modeling:** Mamba blocks capture structural continuity beyond local noise.
- **Convolutional stem:** Helps preserve spatial features under blur or compression.
- **Multi-scale fusion:** Provides redundancy by combining information at different granularities.

These design choices collectively enable the model to generalize well, even under degraded acquisition conditions, which are common in real-world environmental scanning.

## 5 Discussion

The proposed Mamba-integrated deep learning framework demonstrates strong performance in classifying five major categories of airborne particulate pollutants using SEM images, achieving a classification accuracy of 91.8% and a recall of 91.2%. Beyond these specific pollutant categories, our approach has the potential to generalize to broader environmental monitoring scenarios. For instance, similar methodologies could be applied to **soil and water pollution analysis**, where identifying particulate contaminants such as heavy metals, microplastics, or agricultural residues is crucial for ecosystem and human health. By retraining or fine-tuning the model on datasets derived from soil sediments or water filtrates, the framework could be extended to characterize complex pollutant mixtures in diverse ecological contexts.

Furthermore, the integration of **multimodal imaging data**, such as combining SEM with Raman spectroscopy, hyperspectral imaging, or energy-dispersive X-ray spectroscopy (EDS), presents an opportunity to capture complementary structural and compositional information. This multimodal fusion would likely enhance both classification robustness and interpretability, enabling finer discrimination between visually similar but chemically distinct particles.

However, the use of **synthetic data generated by GANs** introduces several important considerations. While GAN augmentation can effectively expand limited training sets and improve model generalization, synthetic images may not fully capture the subtle morphological and textural variations present in real-world samples. This mismatch can potentially lead to overfitting to GAN-specific artifacts or bias the learned feature representations. Additionally, if the GAN is trained on a narrow dataset, the diversity of generated images may be limited, which could reduce the effectiveness of augmentation in practice.

Future research should therefore focus on systematically evaluating the domain gap between real and synthetic data, incorporating adversarial validation or domain adaptation techniques to mitigate distributional discrepancies. Moreover, expanding the dataset across different geographical regions, sampling conditions, and imaging modalities would further enhance the model's robustness and ecological applicability.

## 6 Conclusion

In this paper, we propose a novel deep learning framework for the analysis of air pollutant composition using high-resolution SEM imagery. Our model incorporates structured state-space modeling via Mamba modules, convolutional feature encoding, and gated multi-scale fusion.

To support our study, we first collect a diverse SEM dataset from multiple geographical regions across China. To expand the training dataset, we also proposed to adopt a CGAN to synthesize more samples for training the proposed model to achieve higher classification performance. Experimental results demonstrate that our approach outperforms state-of-the-art convolutional and transformer-based baselines in terms of both accuracy and robustness.

Furthermore, we perform ablation studies to evaluate the contribution of each architectural component and validate the significance of the Mamba mechanism in capturing long-range dependencies in structural morphology. Our robustness analysis confirms that the proposed model maintains high performance even when faced with real-world distortions, highlighting its potential deployment in practical monitoring systems.

In conclusion, this work demonstrates the feasibility and effectiveness of leveraging advanced deep learning architectures like Mamba in environmental forensics, laying the groundwork for intelligent and automated pollution analysis systems. For now, we have not tried the proposed method to analyze other types of pollution. The most challenging part could be gather datasets. Unlike capture visual information in air, it could be more difficult to collect visual data in water or soil. With sufficient data, we believe this method can be generalized to analyze other types of pollution.

## Data Availability

The original contributions presented in the study are included in the article/supplementary material, further inquiries can be directed to the corresponding author.
